# Splicing controls the ubiquitin response during DNA double-strand break repair

**DOI:** 10.1038/cdd.2016.58

**Published:** 2016-06-17

**Authors:** C Pederiva, S Böhm, A Julner, M Farnebo

**Affiliations:** 1 Department of Oncology-Pathology, Cancer Centrum Karolinska (CCK), Karolinska Institutet, Stockholm 17176, Sweden

## Abstract

Although evidence that splicing regulates DNA repair is accumulating, the underlying mechanism(s) remain unclear. Here, we report that short-term inhibition of pre-mRNA splicing by spliceosomal inhibitors impairs cellular repair of DNA double-strand breaks. Indeed, interference with splicing as little as 1 h prior to irradiation reduced ubiquitylation of damaged chromatin and impaired recruitment of the repair factors WRAP53*β*, RNF168, 53BP1, BRCA1 and RAD51 to sites of DNA damage. Consequently, splicing-deficient cells exhibited significant numbers of residual *γ*H2AX foci, as would be expected if DNA repair is defective. Furthermore, we show that this is due to downregulation of the E3 ubiquitin ligase RNF8 and that re-introduction of this protein into splicing-deficient cells restores ubiquitylation at sites of DNA damage, accumulation of downstream factors and subsequent repair. Moreover, downregulation of RNF8 explains the defective repair associated with knockdown of various splicing factors in recent genome-wide siRNA screens and, significantly, overexpression of RNF8 counteracts this defect. These discoveries reveal a mechanism that may not only explain how splicing regulates repair of double-strand breaks, but also may underlie various diseases caused by deregulation of splicing factors, including cancer.

When both strands of DNA are severed simultaneously in close proximity to one another, repair is difficult and genetic information may be lost, making double-strand breaks the most dangerous form of DNA damage. Such damage can be caused by both endogenous (e.g., replication failure) and exogenous factors (e.g., ionizing radiation (IR)) and accurate repair is crucial for maintenance of cellular functions and prevention of diseases, such as cancer and neurodegeneration.^1^ Repair of double-strand breaks by both homologous recombination and non-homologous end joining involves stepwise recruitment of the necessary factors to the site of damage. Such recruitment is facilitated by ubiquitylation of the damaged chromatin by the E3 ubiquitin ligases RNF8 and RNF168 (including histones H2A, H2AX and H1), which enables the accumulation of several downstream repair factors, including 53BP1, BRCA1 and RAD51.^2, 3, 4, 5, 6, 7, 8^ Initial recruitment of RNF8 to double-strand breaks requires interaction with both the anchor protein MDC1 (which must first be phosphorylated by ATM kinase) and the WRAP53*β* protein, which binds MDC1 simultaneously via its WD40 domain, thereby facilitating interaction between RNF8 and MDC1.^2, 3, 4, 5^

The extensive effort focused on identifying the proteins involved in the DNA damage response has revealed that processing and/or splicing of RNA has a key role. Two genome-wide siRNA screens have demonstrated that depletion of various factors involved in these functions results in defective repair of DNA double-strand breaks and consequent genomic instability.^9, 10^ Moreover, in response to DNA damage, numerous proteins involved in RNA processing are phosphorylated by the ATM and ATR kinases.^11^ Together, these observations indicate that splicing proteins have key roles in preserving genomic integrity, but the underlying mechanisms remain largely unknown.

RNA splicing, that is, removal of introns from newly synthesized pre-mRNAs and re-joining of the exons, is carried out by the large macromolecular spliceosome consisting of five small nuclear ribonucleoproteins (referred to as U1, U2, U4, U5 and U6 snRNPs) and numerous associated proteins. The spliceosome is assembled on the nascent pre-mRNA in a stepwise manner beginning with the binding of the U1 and U2 snRNPs. Subsequent recruitment of the U4/U6.U5 tri-snRNP triggers major structural rearrangements that activate the catalytic capacity of the spliceosome (reviewed by Matera and Wang^12^ and Schneider-Poetsch *et al.*^13^).

Various natural compounds inhibit splicing by blocking different steps of spliceosome assembly. For example, the macrolide pladienolide B inhibits splicing factor 3B subunit 1 (SF3B1) of the U2 snRNP^14^ and the biflavonoid isoginkgetin prevents the recruitment of the U4/U6.U5 tri-snRNP.^15^

The significant link between splicing defects and human diseases, including cancer and inherited disorders, together with the recent establishment of splicing as an important aspect of DNA repair and genomic stability, emphasizes the importance of elucidating the underlying mechanism(s). In the current study, we took advantage of the fact that pladienolide B and isoginkgetin rapidly inhibit splicing to distinguish between direct participation of the spliceosome in DNA repair and indirect effects on gene expression that may arise from long-term depletion of splicing factors. This approach revealed that splicing regulates expression of the ubiquitin ligase RNF8 and thereby ubiquitin-signaling at DNA double-strand breaks. These findings provide important insight into the mechanism by which splicing contributes to the repair of such breaks and demonstrates that loss of RNF8 may thus serve as an indicator of defective DNA repair associated with splicing dysfunction.

## Results

### Inhibition of pre-mRNA splicing impairs recruitment of repair proteins to DNA double-strand breaks

**Figure 1:**
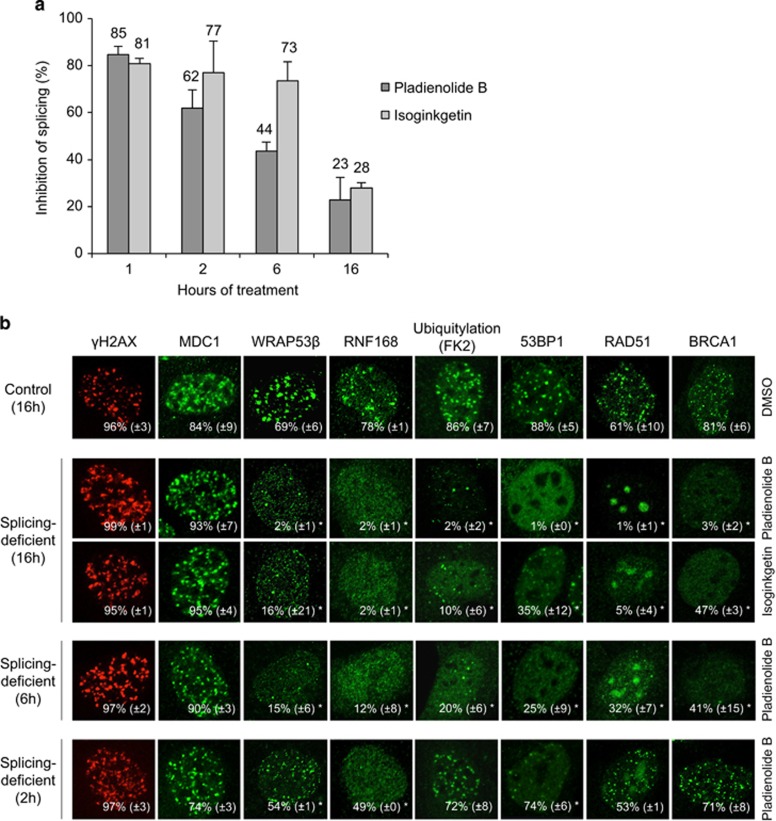
Recruitment of DNA repair factors to double-strand breaks is impaired in splicing-deficient cells. (**a**) HeLa Luc-I or Luc cells were exposed to DMSO (control), 100 nM pladienolide B or 50 *μ*M isoginkgetin for 1–16 h. After normalization to the corresponding DMSO value, the ratio between Luc-I and Luc luciferase activity is presented as a percentage. Means±S.D. are shown, *n*=4. (**b**) U2OS cells were treated with pladienolide B or isoginkgetin for 2, 6 or 16 h, irradiated (6 Gy, 1 h recovery) 1 h prior to termination of the treatment, fixed and immunostained for γH2AX, MDC1, WRAP53*β*, RNF168, conjugated ubiquitin recognized by the FK2 antibody, 53BP1, RAD51 or BRCA1. Nuclei were stained with DAPI in all immunofluorescence experiments. The numbers in white represent the percentage of 100–200 cells counted whose nuclei contained >10 IR-induced foci. Means±S.D. are shown, *n*=3. *P-value<0.05, as determined by a non-paired two-tailed Student's *t*-test. The ‘foci-like' accumulations RAD51 after splicing inhibition in (**b**) are not IR-induced foci, but accumulation of RAD51 in the nucleolus for unknown reasons

Exposure of cells to the small molecules pladienolide B or isoginkgetin for 2–16 h lowered their splicing capacity by as much as 75% (as assessed by HeLa reporter cells carrying a luciferase gene that requires splicing for expression of the protein) (Figure 1a; Supplementary Figures S1a–S1c).^16^ After irradiation of U2OS, HeLa and human fibroblast cells that had been pre-treated with pladienolide B or isoginkgetin for 2–16 h, the accumulation of several repair factors – WRAP53*β*, RNF168, 53BP1, BRCA1 and RAD51 – at double-strand breaks, as well as of ubiquitin conjugates (recognized by the FK2 antibody) formed by RNF168 and RNF8 at these sites, were dramatically lower than in control cells (Figure 1b; Supplementary Figures S1d and e). Strikingly, reduced accumulation of these factors was observed after splicing had been inhibited for only 1–5 h prior to irradiation and the extent of reduction correlated directly with the degree and duration of splicing inhibition (Figures 1a and b; Supplementary Figures S1d and e). In contrast, the upstream repair factors MDC1 and *γ*H2AX accumulated in normal amounts at DNA breaks in these splicing-deficient U2OS, HeLa and fibroblast cells at all time-points (Figure 1b; Supplementary Figures S1d and e). The possibility of indirect effects of splicing inhibition on cell cycle progression was excluded (Supplementary Figure S1f). Thus, inhibition of splicing disrupts the DNA damage response directly and impairs ubiquitin-mediated assembly of repair factors at double-strand breaks in both cancer and non-transformed cells.

### Inhibition of splicing downregulates the expression of repair factors

**Figure 2:**
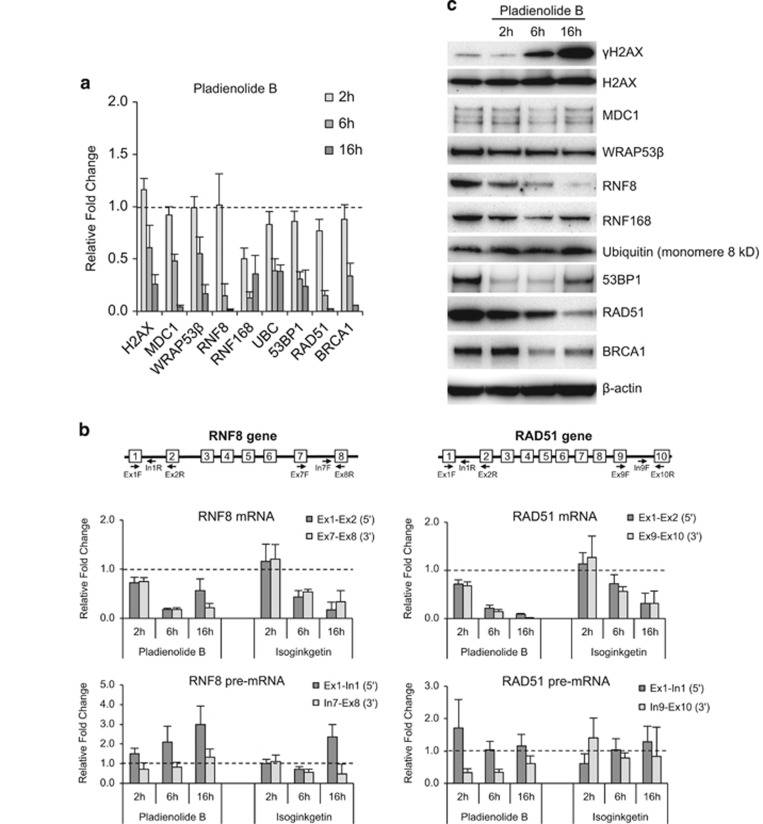
Inhibition of splicing downregulates repair factors at both the mRNA and protein levels. (**a**) U2OS cells were treated with pladienolide B for 2, 6 and 16 h and irradiated (6 Gy, 1 h recovery) 1 h prior to termination of the treatment. The levels of the indicated mRNAs were measured through qPCR analysis. The change is relative to the DMSO control and two reference genes (18S rRNA and *β*-actin). Means±S.D. are shown, *n*=3. (**b**) qPCR analysis of the levels mRNA and pre-mRNA for RNF8 and RAD51 in U2OS cells treated with pladienolide B or isoginkgetin for 2, 6 or 16 h and irradiated (6 Gy, 1 h recovery) 1 h prior to termination of this treatment. The change is expressed relative to the DMSO control value and the levels of mRNA for two reference genes (18S rRNA and *β*-actin). Means±S.D. are shown, *n*=3. The arrows indicate the positioning of the PCR primers used and their sequences are shown in Supplementary Table S1. (**c**) Western blotting following pladienolide B treatment of U2OS cells as described in (**a**). *β*-Actin was used as a loading control. Densitometric quantification of each protein is shown in Supplementary Figure S2B. The three MDC1 bands correspond to the unphosphorylated, phosphorylated and hyperphosphorylated forms of full-length MDC1

To assess whether the attenuated accumulation of repair factors at DNA lesions caused by pladienolide B or isoginkgetin is a consequence of lowered expression of these factors due to impaired splicing of their transcripts, we analyzed the levels of mRNA and protein for H2AX, MDC1, WRAP53*β*, RNF8, RNF168, ubiquitin (UBC), 53BP1, BRCA1 and RAD51 by quantitative (q)PCR and western blotting, respectively. The levels of mRNA were reduced in proportion to the degree and duration of splicing inhibition (Figure 2a; Supplementary Figure S2a), falling by at least 50% after 6 h of treatment, which suggests that their half-lives are this long or shorter.

To determine whether pladienolide B or isoginkgetin reduce levels of mRNA by attenuating splicing or transcription, the levels of the mRNA and pre-mRNA for RNF8 and RAD51 were assessed using different sets of primer for their 5′ and 3′ ends (Figure 2b). Both drugs clearly inhibited splicing as reflected in lower mRNA levels and higher levels of pre-mRNA, but they also appeared to reduce transcription with stalled or reduced production of pre-mRNA at certain time-points, in agreement with the close coupling between transcription and splicing, where ongoing splicing promotes transcription.^17, 18, 19, 20^

In splicing-deficient cells, the level of H2AX protein was unchanged, whereas phosphorylation of this protein (*γ*H2AX) was enhanced, particularly following 6 and 16 h of treatment. This observation is indicative of accumulation of DNA damage, which is consistent with the attenuated assembly of repair proteins at these sites (Figure 2c and Supplementary Figure S2b). The half-life of H2AX (as determined by a cycloheximide chase) is approximately 16 h (Supplementary Figures S2c and d). The level of the MDC1 protein, with a half-life of ~29 h, was also relatively unaltered by inhibition of splicing, as was the level of ubiquitin.

After 16 h of inhibited splicing, the level of WRAP53*β* protein was reduced and the levels of RNF168, RAD51 and BRCA1 severely attenuated. The half-lives of these proteins range from ~8 to 16 h (Figure 2c and Supplementary Figures S2b–d). The level of 53BP1 protein, with half-life of 10 h, was reduced initially, but consistently elevated at the latest time-point examined. The elevated expression of 53BP1 protein at 16 h is puzzling, but may reflect enhanced stability in response to accumulation of DNA breaks or the cell stress induced by the splicing inhibitors. Indeed, the stability of the 53BP1 protein is increased following DNA damage (Supplementary Figures S2c and d).

The level of RNF8 protein, in particular, was reduced following 2 h of inhibited splicing (Figure 2c; Supplementary Figure S2b), and after 16 h, RNF8 was barely detectable, which is consistent with its half-life of approximately 6 h (Supplementary Figures S2c and d).

Measurement of the half-lives of all repair proteins following irradiation revealed that the stability of H2AX, WRAP53*β*, RNF8, RNF168, BRCA1 and 53BP1 was enhanced, while that of MDC1 declined (Supplementary Figures S2c and d).

In itself DMSO, in which both splicing inhibitors were dissolved, exerted no effect on the expression of these repair proteins at either the mRNA or protein level (Supplementary Figures S3a and b). Together, these findings indicate that repair proteins, and in particular RNF8, are short-lived and require ongoing splicing for continued expression.

### Overexpression of RNF8 restores effective repair of double-strand breaks in splicing-deficient cells

**Figure 3:**
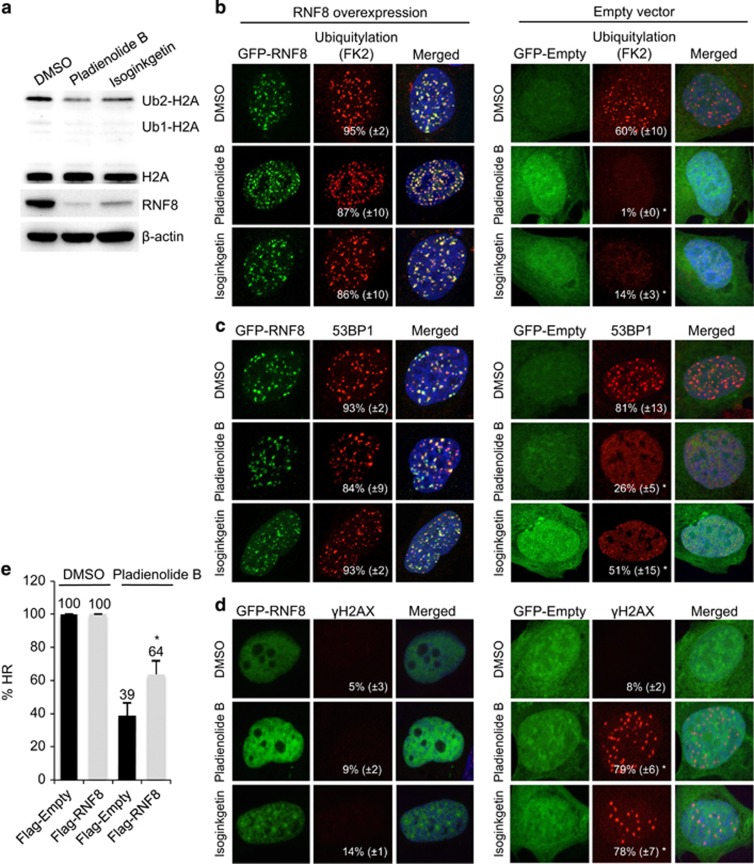
Overexpression of RNF8 restores repair of DNA double-strand breaks in splicing-deficient cells. (**a**) U2OS cells were treated with DMSO, pladienolide B or isoginkgetin for 16 h, irradiated (6 Gy, 1 h recovery) 1 h prior to termination of this treatment, and then subjected to western blotting for H2A, RNF8 and *β*-actin. (**b** and **c**) U2OS cells were transfected with either GFP-RNF8 or GFP-Empty for 2 h, followed by addition of pladienolide B, isoginkgetin or DMSO, incubation for an additional 5 h, irradiation with 6 Gy, fixation 1 h later, and immunostaining for (**b**) conjugated ubiquitin (FK2 antibody) and (**c**) 53BP1. The numbers in white indicate the percentage of 100 transfected, i.e, green cells whose nuclei contained >10 IR-induced foci. Means±S.D. are shown, *n*=3. (**d**) U2OS cells were treated as above, except that fixation was performed 24 h after irradiation and the immunostaining was for *γ*H2AX. Again, the white numbers indicate the percentage of 100 transfected, i.e, green cells whose nuclei contained >10 γH2AX foci. Means±S.D. are shown, *n*=3. (**e**) The efficiency of HR measured in direct repeated-GFP U2OS cells transfected with I-*Sce*I in combination with either Flag-Empty or Flag-RNF8 for 24 h, followed by addition of DMSO or pladienolide B and incubation for another 24 h. Means±S.D. are shown, *n*=3. *P<0.05, as determined by a non-paired two-tailed Student's *t*-test

Assembly of RNF168, 53BP1, BRCA1 and RAD51 depends on ubiquitylation of damaged chromatin by RNF8, the first E3 ligase to localize to DNA breaks,^2, 3, 4, 5, 7^ and reduced expression of RNF8 might be the underlying cause for the defective recruitment of these proteins to DNA breaks in splicing-deficient cells. Indeed, inhibition of splicing attenuated ubiquitylation of H2A, which is known to be a substrate for both RNF8 and RNF168 (Figure 3a).^5, 21^ We could not assess the assembly of endogenous RNF8 protein in repair foci owing to a lack of appropriate antibodies, but monitoring of transiently expressed GFP-RNF8 did reveal accumulation in repair foci in splicing-deficient cells (Figure 3b and Supplementary Figure S4a). Surprisingly, ubiquitylation at sites of DNA damage (as determined by FK2 staining and western blotting for H2A ubiquitylation), and downstream 53BP1 repair foci, previously shown to be sensitive to inhibition of splicing, were restored by overexpression of GFP-RNF8, but not by GFP alone (Figures 3b and c; Supplementary Figure S4b). Moreover, overexpression of GFP-RNF8 restored 53BP1 foci in human fibroblasts after splicing had been inhibited (Supplementary Figure S4c) and, similarly, 53BP1 repair foci were restored in splicing-deficient cells when Flag- or HA-tagged RNF8 was overexpressed (Supplementary Figure S4d).

The accumulation of 53BP1 in repair foci when RNF8 was overexpressed was not due to stabilization of this protein, because overexpression of 53BP1 itself (GFP-53BP1) did not restore accumulation (Supplementary Figure S5a). Moreover, neither overexpression of GFP-MDC1, GFP-RAD51 nor GFP-WRAP53*β* could restore the assembly of repair factors at DNA double-strand breaks (Supplementary Figures S5b and c; data not shown). Overexpression of GFP-RNF168 gave rise to nuclear aggregations in both non-irradiated and irradiated cells, as also observed by others,^22, 23^ and these aggregates were indistinguishable from repair foci (with regards to size and enrichment in repair factors, such as 53BP1 and ubiquitin conjugates) even in non-irradiated cells (Supplementary Figure S5d). Therefore, we could not determine whether upon inhibition of splicing overexpression of RNF168 restored the assembly of repair factors at DNA breaks. Nonetheless, these RNF168 aggregates were not affected by splicing inhibition (Supplementary Figure S5e).

Notably, splicing-deficient cells exhibited significant amounts of residual γH2AX 24 h after irradiation, whereas when overexpressing GFP-RNF8, these cells demonstrated normal clearance of γH2AX, consistent with efficient DNA repair (Figure 3d). Western blotting confirmed that the level of γH2AX in cells overexpressing GFP-RNF8 was reduced 24 h after irradiation (Supplementary Figure S5f). As the expression of downstream repair factors was similar in cells with or without GFP-RNF8 (Supplementary Figure S5f), the possibility that overexpression of RNF8 rescues DNA repair indirectly by restoring this expression was eliminated.

To confirm that overexpression of RNF8 actually restores repair of double-strand breaks in splicing-deficient cells and not only the associated signaling cascade, we examined the efficiency of homologous recombination (HR) in U2OS cells carrying the construct with a direct repeated-GFP sequence. In these cells, expression of exogenous I-*Sce*I introduces a single double-strand break, the repair of which by HR produces a functional coding sequence for GFP.^24, 25^ It is noteworthy that this assay revealed that inhibition of splicing lowered the efficiency of HR by 60% and that simultaneous overexpression of RNF8 restored this repair pathway (Figure 3e). In contrast to the almost complete restoration of repair foci upon overexpression of RNF8 in cells with inhibited splicing, the efficiency of HR repair was only restored partially. This might reflect the fact that the transfection efficiency was not very high (around 20–30%), and for examination of repair foci, the transfected cells could be identified for quantification, whereas in the case of the HR assay, the entire cell population was analyzed. Moreover, with the HR assay, the I-*Sce*I and RNF8 plasmids must be in the same cell for rescue to occur, which further dilutes the number of cells in which rescue can take place.

Altogether, these findings demonstrate that maintenance of RNF8 levels by functional splicing is essential for ubiquitin-dependent repair of double-strand breaks. The level of RNF8 appear to be of particular importance for DNA repair, because other repair factors in the same pathway, although also severely downregulated by inhibition of splicing, could nonetheless function adequately once the RNF8 levels was restored.

### Downregulation of RNF8 underlies the defective DNA repair associated with knockdown of various splicing factors in genome-wide screens

**Figure 4:**
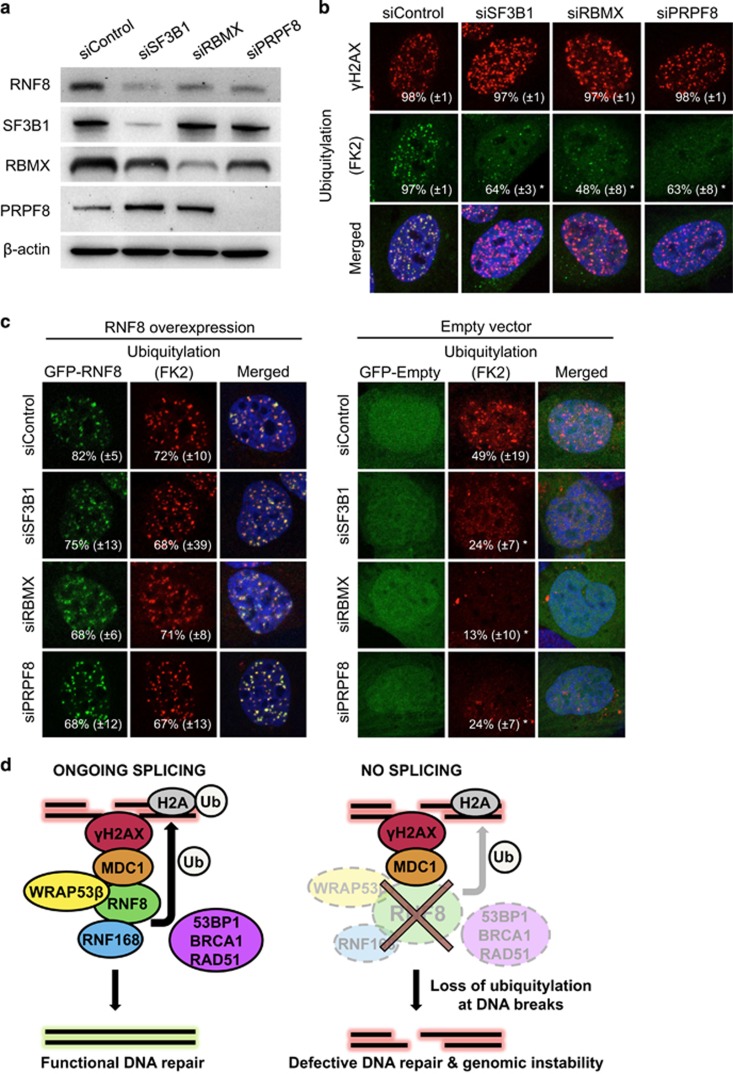
When splicing-related factors are depleted, overexpression of RNF8 restores repair of DNA double-strand breaks. (**a**) Western blotting of SF3B1, RBMX, PRPF8 and RNF8 in U2OS cells treated for 48 h with the siRNAs indicated. *β*-Actin was used as a loading control. (**b**) U2OS cells were transfected with the siRNAs indicated for 48 h, exposed to IR (6 Gy), and 1 h later, immunostained for *γ*H2AX and conjugated ubiquitin (with the FK2 antibody). (**c**) U2OS cells were treated with the siRNAs indicated for 40 h, followed by transfection with either GFP-RNF8 or GFP-Empty plasmid for 7 h; exposure to IR (6 Gy) and fixation 1 h later and immunostaining for conjugated ubiquitin. The white numbers indicate the percentage of 100 transfected cells, i.e, green cells whose nuclei contained >10 IR-induced foci. Means±S.D. are shown, *n*=3. *P<0.05, as determined by a non-paired two-tailed Student's *t*-test. (**d**) Schematic model of how splicing regulates repair of DNA double-strand breaks

Recent genome-wide screens of siRNA demonstrate that depletion of various splicing factors results in defective repair of DNA double-strand breaks and genomic instability.^9, 10^ To examine whether downregulation of RNF8 could be the critical event in this context, we knocked down SF3B1 (component of U2 snRNP), pre-mRNA processing factor 8 (PRPF8) (component of U2 and U12 snRNPs) and RNA binding motif protein, X-linked (RBMX) (regulates alternative splicing), splicing-related proteins whose depletion was associated with defective repair in both screens. In all three cases, we observed the downregulation of RNF8 and significantly attenuated ubiquitylation of damaged chromatin (Figures 4a and b; Supplementary Figures S6a and b), confirming the involvement of these factors in the ubiquitin response critical for repair of DNA double-strand breaks.

Significantly, re-introduction of GFP-RNF8 into these cells restored ubiquitylation at DNA breaks and subsequent recruitment of 53BP1 completely, whereas overexpression of GFP alone did not (Figure 4c; Supplementary Figure S6c). However, the defects in ubiquitylation at DNA breaks caused by knockdown of either WRAP53*β*, recently shown to be essential for accumulation of RNF8 at DNA lesions by scaffolding RNF8-MDC1 interactions,^2^ or RNF168, which catalyzes the formation of poly-ubiquitin chains at sites of DNA damage could not be counteracted by overexpression of GFP-RNF8 (Supplementary Figure S6d). Therefore, we conclude that overexpression of GFP-RNF8 only restores defects in repair caused by downregulation of this protein itself and not defective repair caused by other mechanisms.

## Discussion

Here, we report that functional splicing has a fundamental role in the repair of DNA double-strand breaks. Using small molecules that interfere with assembly of the spliceosome or siRNAs targeting splicing factors, we found that reduced splicing efficiency is associated with attenuated ubiquitylation at sites of DNA damage, which in turn impairs recruitment of repair factors. We provide evidence that this decrease in ubiquitylation is due to downregulation of the E3 ligase RNF8 and re-introduction of this protein restores repair of DNA double-strand breaks. Our current observations thus support a model in which ongoing splicing promotes the expression of several short-lived repair factors, including RNF8, RNF168 and RAD51, where appropriate expression of RNF8 is particularly critical for repair of double-strand breaks (Figure 4d). In this manner, splicing regulates repair of the most dangerous type of DNA damage and is required for genomic stability.

Splicing requires stepwise assembly of the spliceosome on pre-mRNAs and we inhibited this assembly with pladienolide B and isoginkgetin.^14, 15^ Using reporter cell lines,^16^ we showed that splicing was significantly inhibited after only a couple of hours of treatment with these drugs, with pladienolide B being more potent at early time-points. After 16 h, both molecules inhibited cellular splicing capacity by approximately 75%.

Monitoring of the recruitment of repair factors to DNA breaks in splicing-inhibited cells revealed dramatically attenuated accumulation of several such factors, including WRAP53*β*, RNF168, conjugated ubiquitin, 53BP1, RAD51 and BRCA1. Indeed, following exposure to pladienolide B for 16 h prior to irradiation, less than 3% of the cells exhibited formation of repair foci. In fact, with pladienolide B treatment for only 1 h prior to irradiation, the number of cells expressing certain repair factors in repair foci was reduced by as much as 35%, demonstrating that even short-term inhibition of splicing significantly impairs the cellular response to DNA damage. The upstream proteins γH2AX and MDC1 still formed foci in our splicing-inhibited cells at all time-points, suggesting that only factors downstream of MDC1 are sensitive to such inhibition.

The mechanism of splicing-dependent DNA repair was explored further by examining the expression of repair factors. The levels of mRNA encoding all of the repair factors studied were lowered in association with inhibition of splicing, probably reflecting rapid degradation of aberrantly spliced transcripts by the nonsense-mediated decay pathway.^26^ The levels of RNF168 and RAD51 protein were severely reduced and RNF8 protein was barely detectable after 16 h of inhibition, while the levels of H2AX, MDC1, WRAP53*β* and 53BP1 protein were not significantly altered. Apparently, RNF8, RNF168 and RAD51 turnover rapidly and functional splicing of their pre-mRNAs is required for their continuous synthesis. Measurement revealed that the RNF8 protein is the most short-lived of these, with a half-life of approximately 5 h, that is, considerably shorter than the average protein half-life of 20 h or longer.^27, 28^

RNF8 acts immediately downstream of MDC1 in the DNA repair recruitment cascade and its pronounced downregulation by splicing inhibition might be what led to defective repair. In line with this proposal, we consistently observed impairment of DNA repair downstream of MDC1 in connection with splicing inhibition. As we could not monitor endogenous RNF8 protein in repair foci owing to the lack of antibodies that detect such accumulation, we transiently overexpressed RNF8 tagged with GFP, Flag or HA and found that these formed foci at DNA breaks in splicing-deficient cells. Interestingly, such overexpression also fully restored ubiquitylation of damaged chromatin, as well as downstream accumulation of 53BP1, subsequent clearance of γH2AX and repair by HR.

The DNA damage response was not restored by overexpression of MDC1, WRAP53, 53BP1 or RAD51. Moreover, overexpression of 53BP1 itself was not sufficient for it to accumulate in repair foci. Thus, loss of RNF8 appears to result in defective assembly of downstream factors and the level of this protein seems to be rate-limiting for DNA repair, because other repair factors in the same pathway, which are also severely downregulated in association with inhibition of splicing, nonetheless retain their capacity to conduct repair once expression of RNF8 is restored. It remains unclear why the remaining levels of RNF8 are insufficient. Perhaps RNF8 is recruited more strongly for other processes, for example, mitosis.^29^

Although WRAP53*β* acts upstream of RNF8, this protein was unable to form foci in splicing-deficient cells, in agreement with our previous finding that formation of WRAP53*β* foci depends on RNF8 expression (data not shown). The mechanism underlying this co-dependency is at present unclear. It is possible that WRAP53*β* recruits RNF8 to double-strand breaks and that RNF8 in turn promotes retention of WRAP53*β* at this same site by modifying the damaged chromatin. Indeed, a similar co-dependency between RNF8 and the chromatin-remodeling factor CHD4 has been observed, wherein RNF8 targets CHD4 to double-strand breaks and subsequent remodeling of the damaged chromatin by CHD4 is then required for efficient RNF8-mediated ubiquitylation and recruitment of downstream factors to this site.^30^

Recent siRNA screens have highlighted the involvement of pre-mRNA processing in the repair of DNA double-strand breaks.^9, 10^ On the basis of our present findings, we hypothesized that loss of RNF8 could be the primary cause of defective repair. Indeed, knockdown of several splicing-related proteins (SF3B1, RBMX and PRPF8) identified by both screens resulted in defective ubiquitylation at sites of DNA damage and impaired repair and subsequent overexpression of RNF8 restored both ubiquitylation and downstream signaling.

An intriguing alternative mechanism for the repair of double-strand breaks involving RBMX has been proposed. This model includes localization of this protein to DNA lesions and regulation of alternative splicing of BRCA2 pre-mRNA.^9^ Our finding that loss of RBMX also results in defective ubiquitylation at sites of DNA damage due to loss of RNF8 expression reveals an additional role played by RBMX in connection with the repair of double-strand breaks.

It is tempting to speculate that downregulation of RNF8 not only underlies the defective repair connected with loss of splicing-related proteins, but is also involved in human diseases such as retinitis pigmentosa, which is caused by inherited mutations in the gene encoding the splicing factor PRPF8 and associated with severe vision impairment and even blindness.^31, 32^ Our discovery that PRPF8 regulates RNF8-mediated DNA repair not only reveals a novel function of the PRPF8 protein, but also improves our understanding of the molecular mechanisms underlying this disease. Moreover, this suggests new therapeutic approaches, for example, by restoring RNF8 function in retinal cells deficient in PRPF8. However, this remains to be proven.

In addition, splicing factors are also commonly mutated in various types of cancer. For example, the SF3B1 protein is mutated both in chronic lymphocytic leukemia and myelodysplastic syndrome,^33, 34^ and it is presently unclear how loss of this function promotes tumor initiation/progression. Our observation that loss of SF3B1, as well as general splicing deficiency, impairs DNA repair through downregulation of RNF8 suggests that the level of this latter protein could serve as an indicator of whether the DNA repair in these tumors is defective. Such knowledge could facilitate the choice of treatment, because tumors with altered responses to DNA damage can be either hypersensitive or resistant to genotoxic drugs.

Moreover, inhibition of splicing has antitumoral effects and our current findings shed new light on the underlying mechanism, perhaps helping to improve treatment by indicating that splicing inhibitors and DNA-damaging drugs in combination may kill tumor cells more efficiently.

## Materials and Methods

### Cell lines, culture conditions and treatments

U2OS, HeLa and HeLa Luc/Luc-I cells were cultured in low glucose Dulbecco's modified Eagle medium (HyClone, Thermo Scientific, Stockholm, Sweden) supplemented with 10% fetal bovine serum (Gibco, Thermo Scientific, Stockholm, Sweden) and 2.5 *μ*g/ml plasmocin (InvivoGen, Toulouse, France) at 37 °C in a humidified incubator under 5% CO_2_. Direct repeated-GFP U2OS cells were cultured in high glucose Dulbecco's modified Eagle medium (HyClone) supplemented with 10% fetal bovine serum and 2.5 *μ*g/ml plasmocin at 37 °C in a humidified incubator under 5% CO_2_. Human GM00730 fibroblasts (Coriell Institute, Camden, NJ, USA) were cultured in minimum essential medium supplemented with 15% fetal bovine serum and 2.5 *μ*g/ml plasmocin at 37 °C in a humidified incubator under 5% CO_2_.

For treatment, 100 nM pladienolide B (Santa Cruz Biotechnologies, Heidelberg, Germany) or 50 *μ*M isoginkgetin (Merck Millipore, Solna, Sweden) (final concentrations) were added to the culture medium 1–24 h prior to irradiation or harvesting, with the control cells receiving an equal volume of DMSO (Sigma-Aldrich, Stockholm, Sweden) as the volume of isoginkgetin used. Cycloheximide was added directly to the culture medium to give a final concentration of 50 *μ*g/ml.

### Luciferase splicing assay

The Bright Glo luciferase assay was performed in accordance with the manufacturer's recommendations (Promega, Nacka, Sweden) using the HeLa Luc and Luc-I reporter cell lines.^16^ Light emission was monitored with a Centro LB 960 microplate luminometer (Berthold Technologies, Bad Wildbad, Germany). In brief, this assay is based on HeLa cells stably transfected with the firefly luciferase gene in which the open reading frame is interrupted by an intron (Luc-I) designed to be spliced out with optimal efficiency (Supplementary Figure S1A). Failure to splice out this intron results in a truncated luciferase protein with no activity. To confirm that the changes in Luc-I activity reflect lack of splicing rather than interference with other processes such as transcription or translation, HeLa cells stably expressing the firefly luciferase gene without an intron (Luc) was used in parallel. The luciferase protein from both reporters is short-lived and does not accumulate in cells, allowing measurement of rapid changes in splicing (Supplementary Figures S1a–S1c).

### Irradiation

*γ*-Irradiation with a ^137^Cs source at a photon dose rate of 0.5 Gy/min (Scanditronix, Uppsala, Sweden) was performed at the Karolinska Institutet, Stockholm. Dosimetry was carried out in an ionization chamber with ferrosulfate.

### Immunfluorescent microscopy

Cells were grown on sterilized cover slips, fixed with 4% paraformaldehyde for 15 min (min) at room temperature, permeabilized with 0.1% Triton X-100 for 5 min at room temperature and blocked for 30 min in blocking buffer (2% BSA, 5% glycerol, 0.2% Tween20, 0.1% NaN_3_). Thereafter, these cover slips were incubated for 1 h with primary antibody and 40 min with secondary antibody, both diluted in blocking buffer. After mounting the cover slips with Vectashield mounting medium containing DAPI (Vector Laboratories, Bionordika, Stockholm, Sweden), images were acquired with an LSM700 confocal microscope (Zeiss, Stockholm, Sweden) mounted on the Zeiss Axio observer.Z1 equipped with Plan-Apochromat × 63/1.4 oil immersion lenses, and subsequently processed utilizing Zen 2012 Black (Zeiss).

Pre-extraction: To visualize IR-induced foci containing WRAP53*β*, cells grown on cover slips were first washed with PBS, incubated for 3 min at room temperature with cytoskeleton buffer (CSK) (10 mM Pipes, pH 7.0, 100 mM NaCl, 300 mM sucrose, 3 mM MgCl_2_ and 0.7% Triton X-100), washed with PBS and then incubated for another 3 min with CSK buffer supplemented with 0.3 mg/ml RNase A (CSK+R) and thereafter washed once again with PBS and fixed in 4% paraformaldehyde.

### Cell cycle analysis

For the analysis of the cell cycle, cells were first treated with DMSO, isoginkgetin or pladienolide B for 16 h, then harvested with trypsin, washed in PBS and fixed in 60% ethanol overnight at 4 °C. After removal of the ethanol, the samples were incubated with a solution of RNase A/propidium iodide for 30 min at 37 °C and subsequently analyzed with a NovoCyte apparatus (ACEA Biosciences, San Diego, CA, USA).

### Western blotting

First, the cells were harvested, washed and lysed in ice-cold western blot buffer (100 mM Tris-HCL, pH 8, 150 mM NaCl, 1% NP-40, 1% protease inhibitor cocktail) for 30 min on ice. These lysates were then centrifuged at 14 000 r.p.m. for 15 min at 4 °C and the supernatant assayed for protein concentrations by the Bradford procedure (Bio-Rad, Sundbyberg, Sweden), followed by western blotting using standard procedures.

### Antibodies

The following antibodies were utilized for immunofluorescent staining and western blotting: mouse *α*-*γ*H2AX (cat. no. 05-636, Millipore), rabbit *α*-*γ*H2AX (cat. no. 2577, Cell Signaling, Bionordika, Stockholm, Sweden), mouse *α*-MDC1 (cat. no. ab50003, Abcam, Cambridge, UK), mouse *α*-WDR79 (clone 1F12; used for immunofluorescent staining; Abnova, cat. no. 14761-1-AP, VWR International, Stockholm, Sweden), rabbit *α*-WRAP53-C2 (cat. no. PA-2020-100, Innovagen AB, Lund, Sweden), mouse *α*-RNF8 (cat. no. sc-271462, Santa Cruz Biotechnology), rabbit *α*-RNF168 (cat. no. ABE367, Millipore), mouse *α*-ubiquitin (FK2) (cat. no. ST1200, Calbiochem, Millipore), rabbit *α*-53BP1 (cat. no. NB100-904, Novus Biologicals, Bio-Techne, Abingdon, UK), mouse *α*-BRCA1 (cat. no. sc-6954, Santa Cruz Biotechnology), rabbit *α*-RAD51 (cat. no. ABE257, Millipore), mouse *α*-Ubiquitin (cat. no. sc-8017, Santa Cruz Biotechnology), rabbit *α*-H2A (cat. no. ab18255, Abcam), rabbit *α*-H2AX (cat. no. ab11175, Abcam), rabbit *α*-GFP (cat. no. ab290, Abcam), mouse *α*-HA (cat. no. 23675, Cell Signaling), mouse *α*-*β*-actin (cat. no. A5441, Sigma-Aldrich), rabbit *α*-SF3B1 (cat no. ab39578, Abcam), rabbit *α*-RBMX (cat. no. sc-48796, Santa Cruz Biotechnology) and mouse *α*-PRPF8 (cat.no. ab51366, Abcam). The secondary antibodies were: goat α-rabbit HRP-conjugated (cat. no. 7074, Cell Signaling), horse *α*-mouse HRP-conjugated (cat. no. 7076, Cell Signaling), goat *α*-rabbit Alexa Fluor 488 (cat. no. A11008, Invitrogen, Thermo Scientific, Stockholm, Sweden), goat *α*-mouse Alexa Fluor 488 (cat. no. A11029, Invitrogen), donkey *α*-mouse Alexa Fluor 594 (cat. no. A21203, Invitrogen) and donkey *α*-rabbit Alexa Fluor 594 (cat. no. A21207, Invitrogen).

### RNA extraction and quantification

Cells were lysed in TRIzol (Thermo Scientific) and RNA then extracted with the RNeasy Mini Kit (cat. no. 74106, Qiagen, Sollentuna, Sweden) in accordance with the supplier's recommendations. During the extraction, contaminating DNA was removed by in-column DNase treatment (RNase-Free DNase Set, cat. no. 79254, Qiagen). The final RNA concentration was determined using a Nanodrop 1000 (Thermo Scientific).

### qPCR analysis

cDNA was generated using SuperScript III reverse transcriptase (cat. no. 18080-044, Invitrogen, Thermo Scientific), random hexamer primers (cat. no. SO142, Thermo Scientific), 10 nM dNTPs Mix (cat. no. R0192, Thermo Scientific) and RNaseOUT to inhibit ribonucleases (cat. no. 10777-019, Invitrogen) in accordance with the manufacturer's instructions. qPCR assays were performed in a 7900HT Fast Real-Time PCR System (Applied Biosystems, Thermo Scientific) on MicroAmp Optical 384-well plates (Applied Biosystems). Each assay contained 2X SYBR Green PCR Master Mix (cat. no. 4309155, Applied Biosystems), 250 nM each of the forward and reverse primers (synthesized by IDT, Leuven, Belgium) (Supplementary Table S1) and 12.5 ng cDNA. The relative levels of mRNA were quantified by the comparative Cq procedure (ΔΔCq) and each value then related to the corresponding mock condition as well as to the RNA values of the references *β*-actin and 18S ribosomal RNA.

### Plasmid transfection

Transfections with plasmid were performed using Lipofectamine 2000 (cat. no. 11668-019, Life Technologies, Thermo Scientific) in accordance with the manufacturer's recommendations and using the expression vectors GFP-Empty, GFP-RNF8, GFP-53BP1, GFP-WRAP53*β*, GFP-MDC1, GFP-RAD51, HA-Empty, HA-RNF8, Flag-Empty and Flag-RNF8 as described previously.^2^

### HR assay

U2OS direct repeated-GFP cells (150 000) were seeded into each well of six-well plates and 24 h later co-transfected with an I-*Sce*I vector and either Flag-Empty or Flag-RNF8 for 24 h more, followed by addition of DMSO or pladienolide B and incubation for an additional 24 h. The sells were then harvested, washed in PBS and resuspended in PBS supplemented with 10% fetal bovine serum and the GFP signal arising from the recombination event detected by flow cytometry on a LSRII apparatus (Becton Dickinson, Erembodegem, Belgium). The frequency of HR repair in pladienolide B-treated cells transfected with Flag-Empty or Flag-RNF8 was calculated relative to the corresponding value for DMSO-treated cells.

### siRNA transfection

siRNA (25 nM) targeting SF3B1 (cat. no. D-020061-01-0005, Dharmacon), RBMX (cat. no. D-011691-01-0005, Dharmacon, Karlskoga, Sweden), PRPF8 (cat. no. D-012252-01-0005, Dharmacon) or 10 nM siRNA targeting WRAP53*β* (cat. no. SI00388948, Qiagen), RNF168 (cat. no. SI04143251, Qiagen) or the scramble control (cat. no. 1027280, Qiagen) were transfected into cells for 48 h using HiPerfect transfection reagent (Qiagen) in accordance with the supplier's recommendations.

### Data analysis

Densitometry on western blots was performed with ImageJ 1.49. For evaluation of protein turnover using cycloheximide, densitometric quantification of each protein was normalized to the corresponding value at 0 h and then fitted to a one-phase exponential decay curve using Gnuplot 5.0. Statistical analysis with non-paired two-tailed Student's *t*-tests was carried out in Microsoft Excel 14.4.5 with *P*-values<0.05 being considered significant.

## Abbreviations

HR: homologous recombination
NHEJ: non-homologous end joining
IR: ionizing irradiation
FK2: antibody clone recognizing K^29^-, K^48^- and K^63^-linked polyubiquitinylated and monoubiquitinylated proteins
DMSO: dimethyl sulfoxide

## Supplementary Material

Supplementary Informations
