# Supplementary material for: Splicing controls the ubiquitin response during DNA double-strand break repair
Source: Cell Death Differ. 2016 Jun 17;23(10):1648–57. doi: 10.1038/cdd.2016.58 (PMC5041194; doi:10.1038/cdd.2016.58)
Supplement: Supplementary Informations [file cdd201658x1.pdf]

SUPPLEMENTAL INFORMATION FOR

Splicing controls the ubiquitin response during DNA double-strand break repair

Chiara Pederiva[1,2], Stefanie Böhm[1,2], Alexander Julner[1] and Marianne Farnebo[1,*]

This supplementary information contains:

Figures S1-S6

Figure Legends S1-S6

Table S1

Figure S1

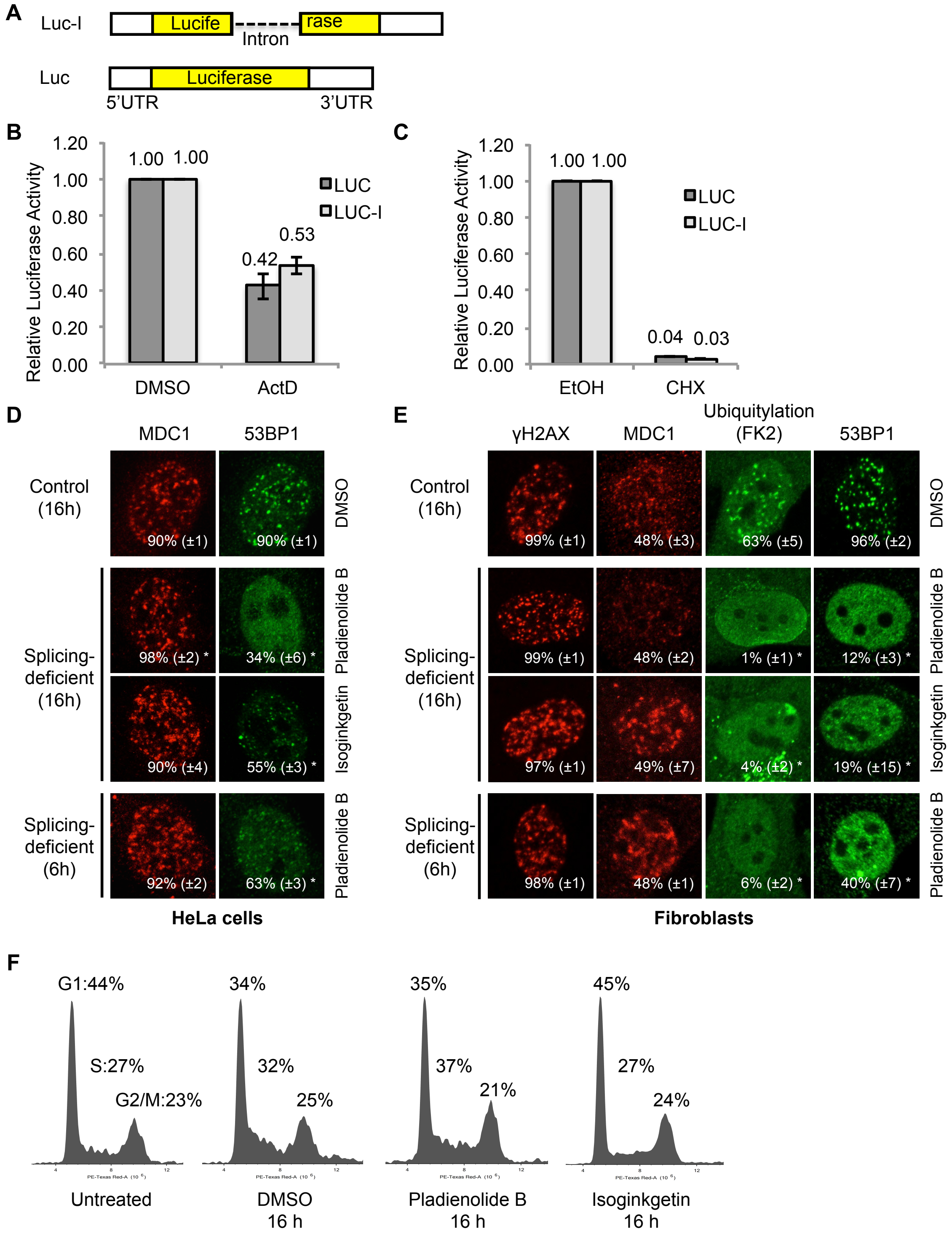
A
Luc-I
Lucife
Intron
rase
Luc
Luciferase
5'UTR
3'UTR
B
Relative Luciferase Activity
1.20
1.00
0.80
0.60
0.40
0.20
0.00
1.00 1.00
0.42
0.53
LUC
LUC-I
DMSO
ActD
C
Relative Luciferase Activity
1.00 1.00
0.04 0.03
LUC
LUC-I
EtOH
CHX
D
MDC1
53BP1
Control (16h)
DMSO
90% (±1)
90% (±1)
Splicing-deficient (16h)
Pladienolide B
98% (±2) *
34% (±6) *
Isoginkgetin
90% (±4)
55% (±3) *
Splicing-deficient (6h)
Pladienolide B
92% (±2)
63% (±3) *
HeLa cells
E
γH2AX
MDC1
Ubiquitylation (FK2)
53BP1
Control (16h)
DMSO
99% (±1)
48% (±3)
63% (±5)
96% (±2)
Splicing-deficient (16h)
Pladienolide B
99% (±1)
48% (±2)
1% (±1) *
12% (±3) *
Isoginkgetin
97% (±1)
49% (±7)
4% (±2) *
19% (±15) *
Splicing-deficient (6h)
Pladienolide B
98% (±1)
48% (±1)
6% (±2) *
40% (±7) *
Fibroblasts
F
G1:44%
S:27%
G2/M:23%
Untreated
34%
32%
25%
DMSO 16 h
35%
37%
21%
Pladienolide B 16 h
45%
27%
24%
Isoginkgetin 16 h

Figure S2

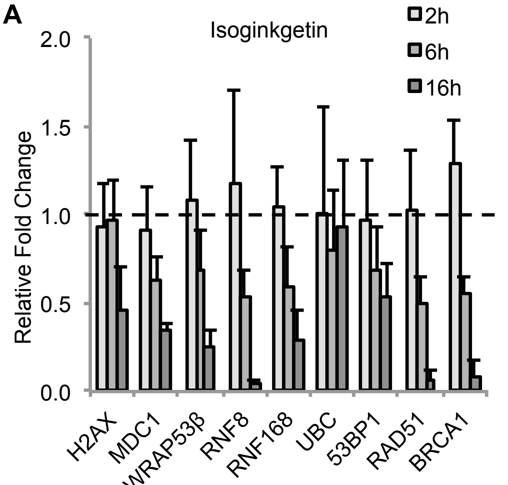

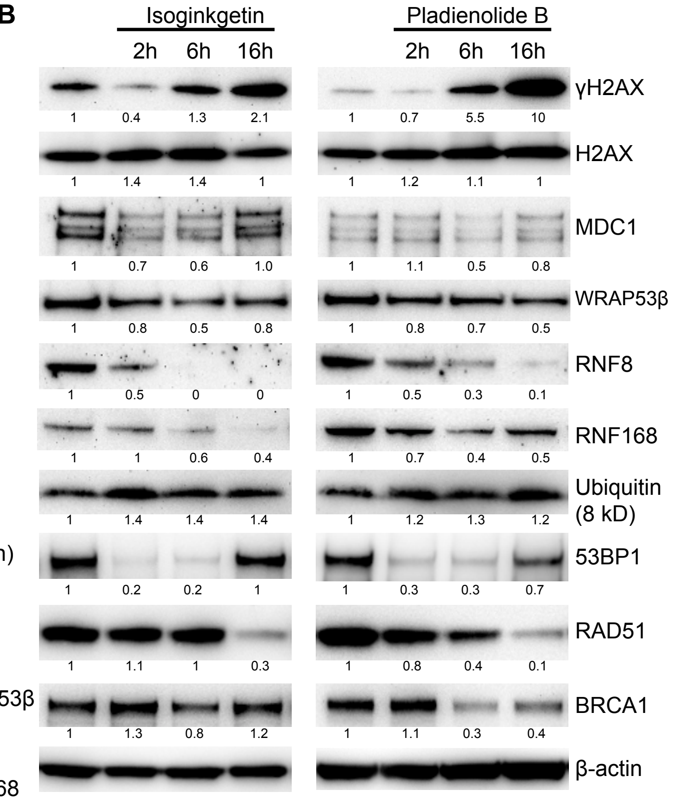

Same blot as Figure 2C

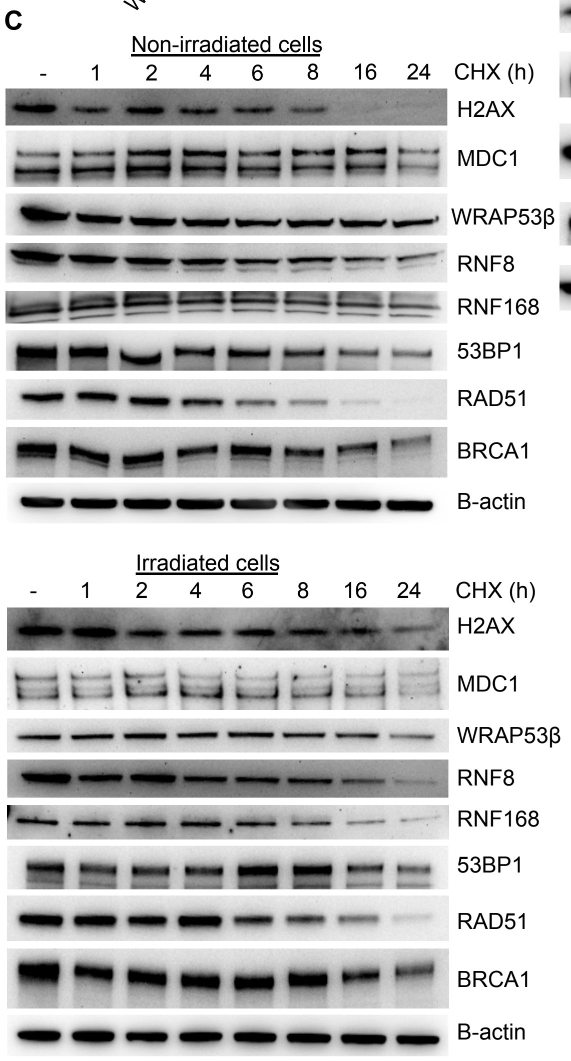

D

Protein Half-Life (hours)		
**Protein**	**No IR**	**IR**
H2AX	16.0 (±9.1)	24.6 (±11.3)
MDC1	29.2 (±14.9)	21.4 (±9.1)
WRAP53	15.8 (±6.0)	18.2 (±6.5)
RNF8	6.1 (±2.0)	7.6 (±2.3)
RNF168	15.3 (±4.8)	16.9 (±9.1)
53BP1	10.0 (±6.0)	26.1 (±11.6)
RAD51	8.1 (±0.5)	8.2 (±1.2)
BRCA1	10.2 (±3.2)	14.0 (±5.7)

Figure S3

A

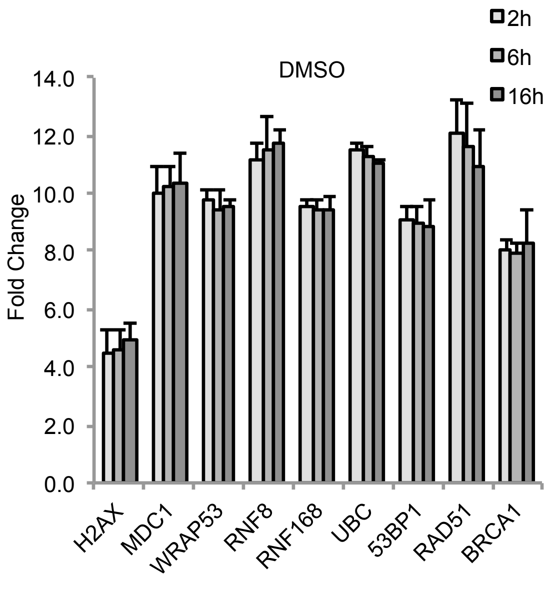
DMSO
2h
6h
16h
14.0
12.0
10.0
8.0
6.0
4.0
2.0
0.0
Fold Change
H2AX
MDC1
WRAP53
RNF8
RNF168
UBC
53BP1
RAD51
BRCA1

B

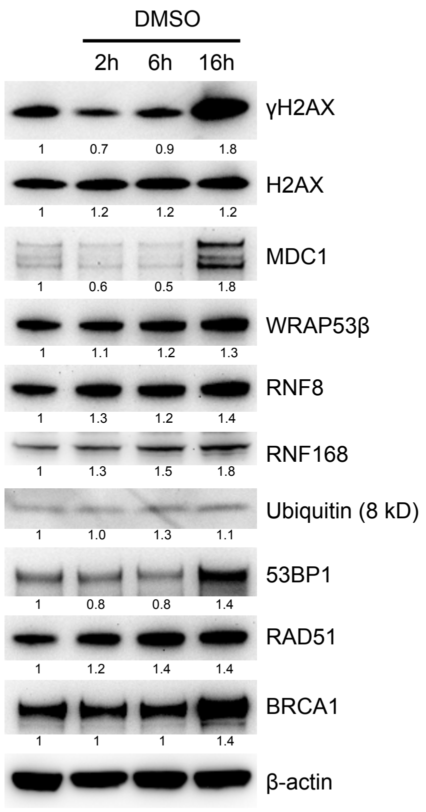
DMSO
2h 6h 16h
γH2AX
1 0.7 0.9 1.8
H2AX
1 1.2 1.2 1.2
MDC1
1 0.6 0.5 1.8
WRAP53β
1 1.1 1.2 1.3
RNF8
1 1.3 1.2 1.4
RNF168
1 1.3 1.5 1.8
Ubiquitin (8 kD)
1 1.0 1.3 1.1
53BP1
1 0.8 0.8 1.4
RAD51
1 1.2 1.4 1.4
BRCA1
1 1 1 1.4
β-actin

Figure S4

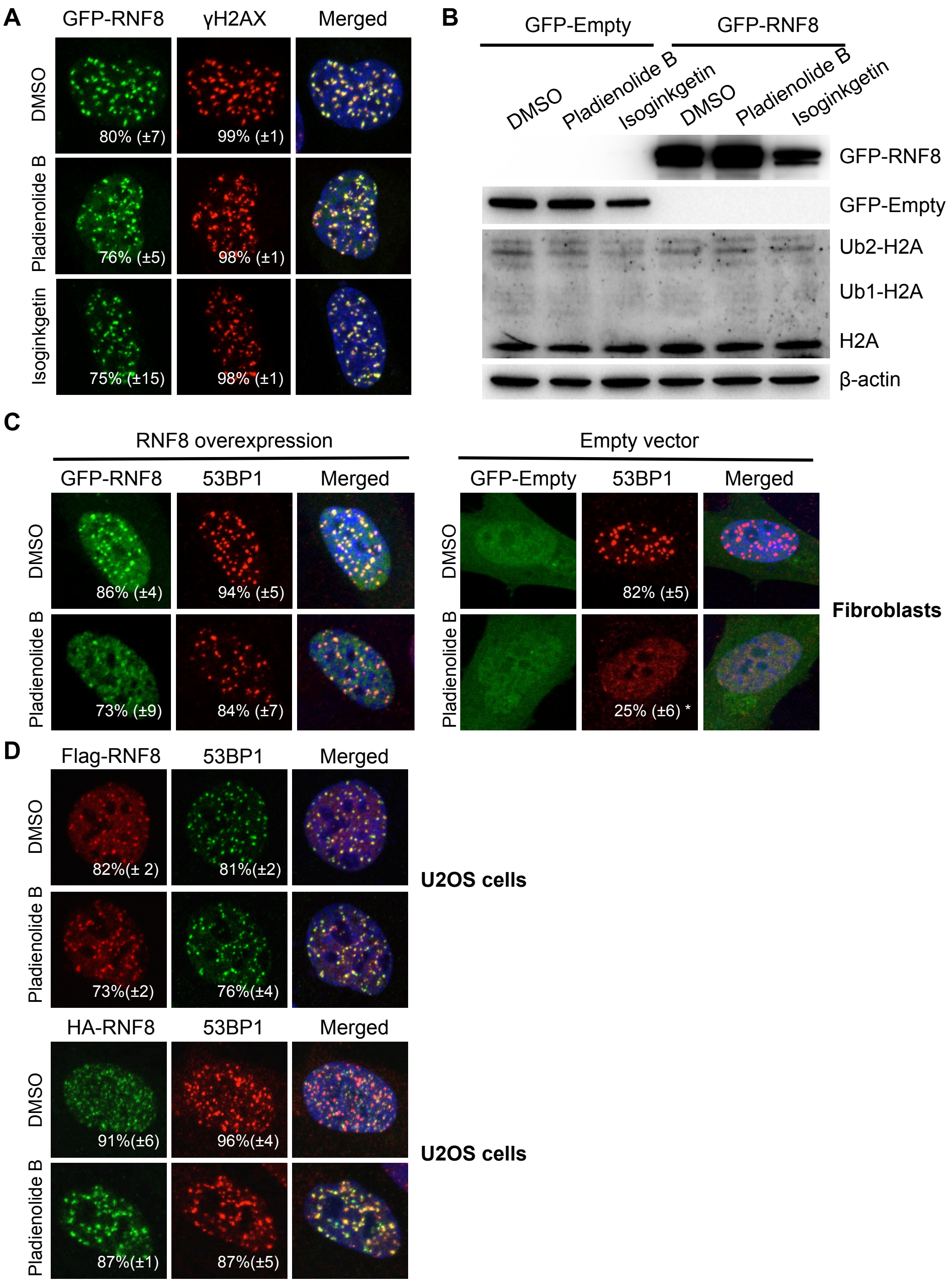

A
GFP-RNF8
γH2AX
Merged
DMSO
Pladienolide B
Isoginkgetin
80% (±7)
99% (±1)
76% (±5)
98% (±1)
75% (±15)
98% (±1)
B
GFP-Empty
GFP-RNF8
DMSO
Pladienolide B
Isoginkgetin
DMSO
Pladienolide B
Isoginkgetin
GFP-RNF8
GFP-Empty
Ub2-H2A
Ub1-H2A
H2A
β-actin
C
RNF8 overexpression
Empty vector
GFP-RNF8
53BP1
Merged
GFP-Empty
53BP1
Merged
DMSO
Pladienolide B
86% (±4)
94% (±5)
82% (±5)
73% (±9)
84% (±7)
25% (±6) *
Fibroblasts
D
Flag-RNF8
53BP1
Merged
DMSO
Pladienolide B
82%(± 2)
81%(±2)
73%(±2)
76%(±4)
U2OS cells
HA-RNF8
53BP1
Merged
DMSO
Pladienolide B
91%(±6)
96%(±4)
87%(±1)
87%(±5)
U2OS cells

Figure S5

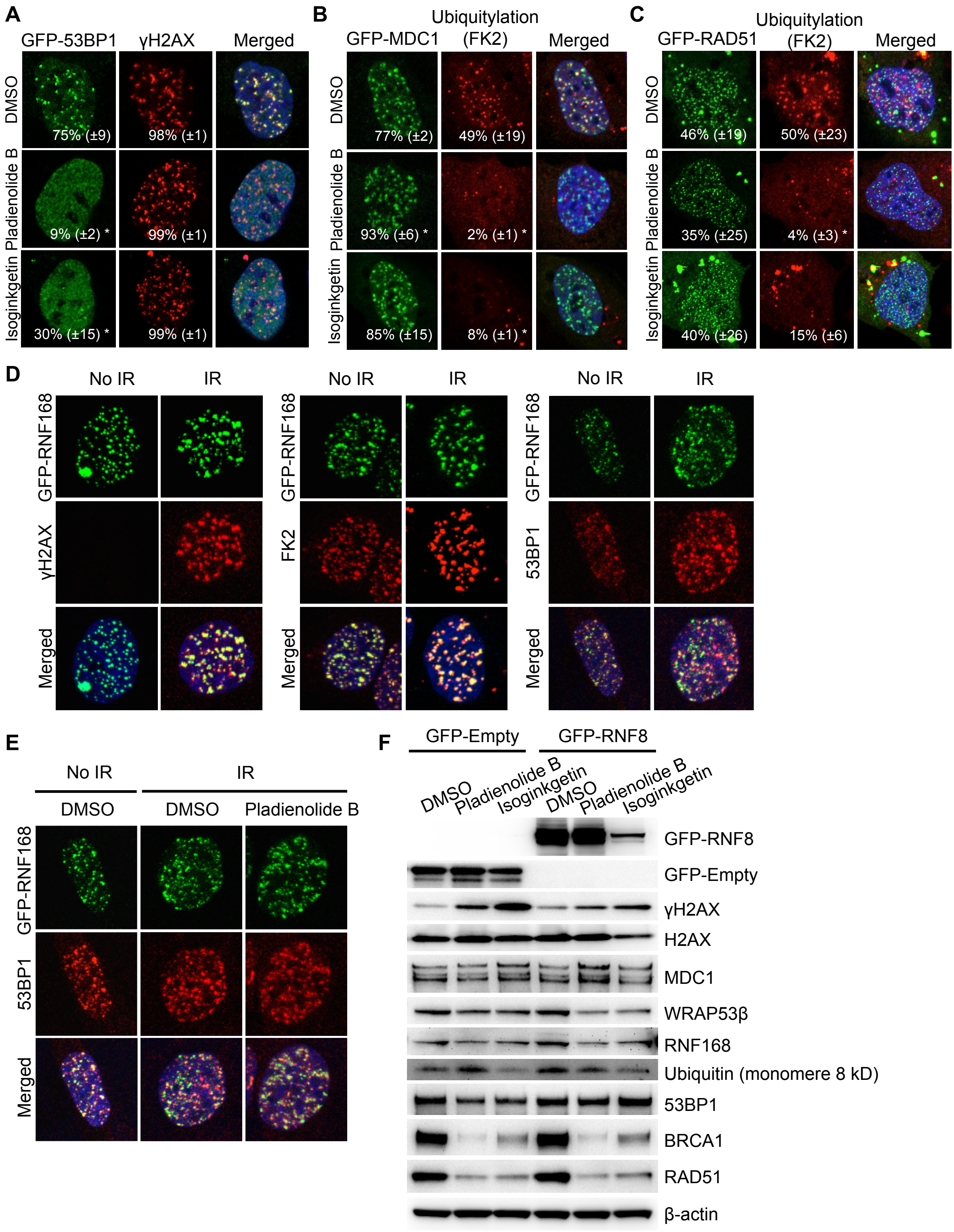
A
GFP-53BP1
γH2AX
Merged
DMSO
Pladienolide B
Isoginkgetin
75% (±9)
98% (±1)
9% (±2) *
99% (±1)
30% (±15) *
99% (±1)
B
GFP-MDC1
Ubiquitylation (FK2)
Merged
77% (±2)
49% (±19)
93% (±6) *
2% (±1) *
85% (±15)
8% (±1) *
C
GFP-RAD51
Ubiquitylation (FK2)
Merged
46% (±19)
50% (±23)
35% (±25)
4% (±3) *
40% (±26)
15% (±6)
D
No IR
IR
GFP-RNF168
γH2AX
FK2
53BP1
Merged
E
No IR
IR
DMSO
Pladienolide B
GFP-RNF168
53BP1
Merged
F
GFP-Empty
GFP-RNF8
DMSO
Pladienolide B
Isoginkgetin
GFP-RNF8
GFP-Empty
γH2AX
H2AX
MDC1
WRAP53β
RNF168
Ubiquitin (monomere 8 kD)
53BP1
BRCA1
RAD51
β-actin

Figure S6

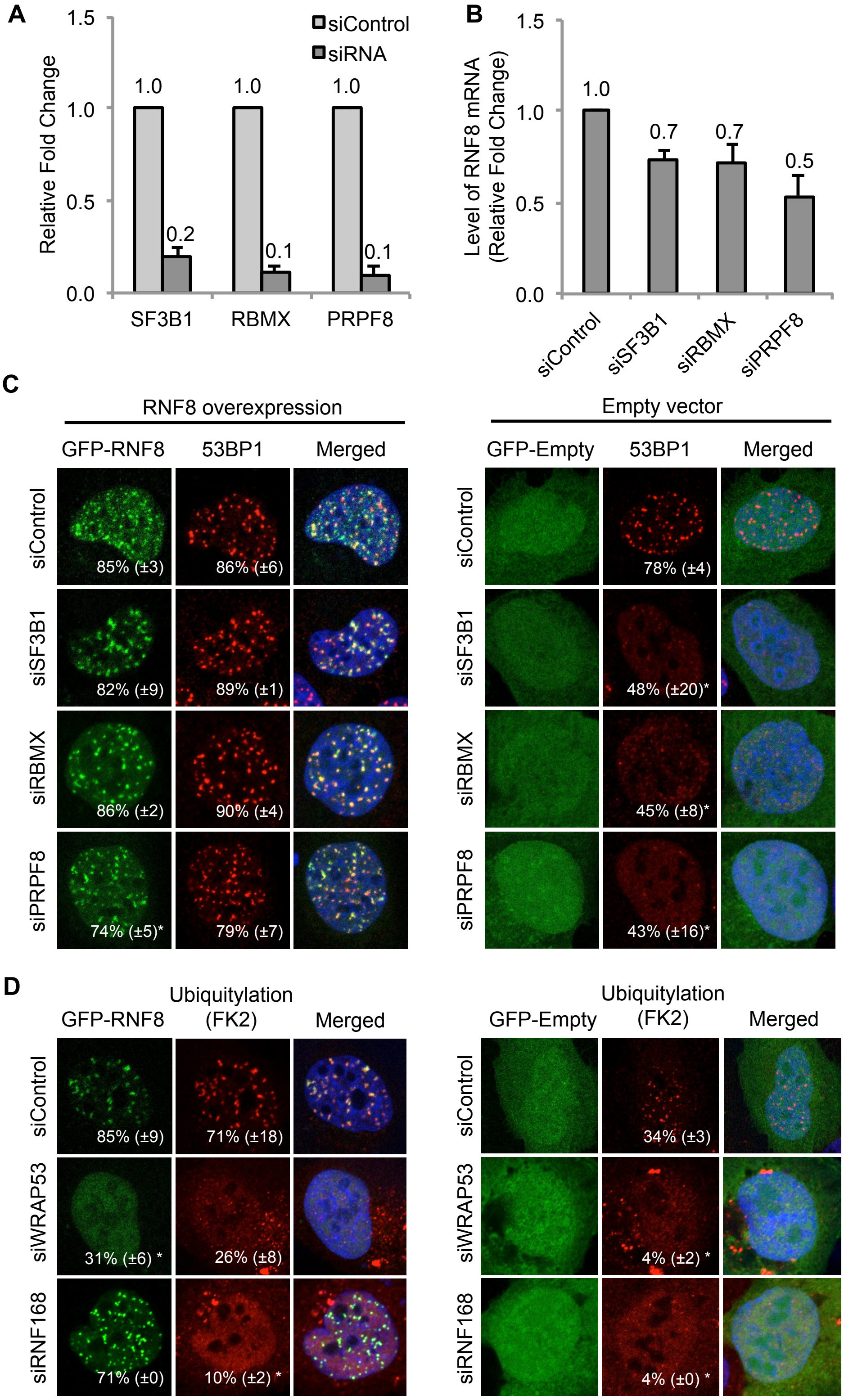
A
Relative Fold Change
1.5
1.0
0.5
0.0
siControl
siRNA
1.0
0.2
1.0
0.1
1.0
0.1
SF3B1
RBMX
PRPF8
B
Level of RNF8 mRNA
(Relative Fold Change)
1.0
0.7
0.7
0.5
siControl
siSF3B1
siRBMX
siPRPF8
C
RNF8 overexpression
GFP-RNF8
53BP1
Merged
85% (±3)
86% (±6)
82% (±9)
89% (±1)
86% (±2)
90% (±4)
74% (±5)*
79% (±7)
Empty vector
GFP-Empty
53BP1
Merged
78% (±4)
48% (±20)*
45% (±8)*
43% (±16)*
D
GFP-RNF8
Ubiquitylation
(FK2)
Merged
85% (±9)
71% (±18)
31% (±6) *
26% (±8)
71% (±0)
10% (±2) *
GFP-Empty
Ubiquitylation
(FK2)
Merged
34% (±3)
4% (±2) *
4% (±0) *
siControl
siSF3B1
siRBMX
siPRPF8
siWRAP53
siRNF168

Supplementary Figure Legends

Figure S1. The effect of pladienolide B and isoginkgetin on the formation of repair foci in HeLa cells and fibroblasts and on the cell cycle

(A) Schematic illustration of the luciferase reporter vectors containing (Luc-I) or lacking (Luc) the intron.

(B and C) The splicing-reporters give rise to unstable mRNAs and proteins that do not accumulate and that respond rapidly to inhibitors of gene and protein expression. Treatment of the Luc and Luc-I cells with (B) 10 ug/ml actinomycin D (ActD) in dimethyl sulfide (DMSO) for 2 h to block transcription or (C) 50 ug/ml cycloheximide (CHX) in ethanol (EtOH) for 4 h to block translation revealed that the luciferase mRNA has a half-life of 2 h and that the levels of the corresponding protein is > than 95% lower following inhibition of protein synthesis for 4 h. Means ± SD are shown, n=4.

(D and E) HeLa cells and human fibroblasts were treated with DMSO, pladienolide B or isoginkgetin for 6 or 16 h, irradiated (6 Gy, 1h recovery) one h prior to termination of the treatment, fixed and immunostained for the indicated repair factors. The white numbers indicate the percentage of 100-200 cells whose nuclei contained >10 IR-induced foci. Means ± SD are shown, n=3. * $p < 0.05$, as determined by a non-paired two-tailed Student's *t*-test.

(F) Cells were either left untreated or treated with DMSO, pladienolide B or isoginkgetin for 16 h, harvested, stained with propidium iodide and subjected to flow cytometry. The numbers indicate the % of cells in each cell cycle phase. Percentages of sub-G1 and super-G2 are not shown.

Figure S2. Inhibition of splicing downregulates repair factors at both the mRNA and protein levels

(A and B) U2OS cells were treated with isoginkgetin for 2, 6 and 16 h, with irradiation (6 Gy, 1 h recovery) one h prior to termination of treatment. (A) qPCR analysis of mRNA levels after isoginkgetin treatment. The change is relative to the DMSO control and two reference genes (18S rRNA and β-actin). Means ± SD are shown, n=3. (B) Western blotting following isoginkgetin and pladienolide B treatment. β-actin was used as a loading control. The numbers in black represent densitometric quantification of each protein after normalization to the corresponding β-actin value for each time point and to its own protein value in untreated cells (first lane). The western blot of pladienolide B-treated cells is the same blot as previously shown in Figure 2C but now including quantifications.

(C) Representative western blots of protein levels in non-irradiated or irradiated U2OS cells treated with cycloheximide (CHX, 50 µg/ml) for the periods indicated. In the case of irradiation, cells were irradiated immediately before addition of cycloheximide to the cell media.

(D) Table of protein half-lives in non-irradiated or irradiated U2OS cells. To monitor protein turnover, non-irradiated or irradiated U2OS cells were treated with cycloheximide (50 µg/ml) for the periods indicated. Protein levels were quantified by image densitometry and after normalization to the corresponding protein value at 0 h, the half-life of each protein was calculated from fitted one-phase exponential decay curves. Means ± SD are shown, n≥3.

Figure S3. DMSO treatment does not alter protein or RNA levels of repair factors

(A and B) U2OS cells were treated with DMSO for 2, 6 and 16 h, with irradiation (6 Gy, 1 h recovery) one h prior to termination of treatment. (A) qPCR analysis of mRNA levels after DMSO treatment. The change is relative two reference genes (18S rRNA and β-actin). Means

± SD are shown, n=3. (B) Western blotting following DMSO treatment. β-actin was used as a loading control. The numbers in black represent densitometric quantification of each protein after normalization to the corresponding β-actin value for each time point and to its own protein value in untreated cells.

Figure S4. Overexpression of RNF8 restores repair of DNA double-strand breaks in splicing-deficient cells

(A) U2OS cells were transfected with GFP-RNF8 for 2 h, followed by addition of pladienolide B, isoginkgetin or DMSO, incubation for an additional 5 h, irradiation with 6 Gy, fixation 1 h later and immunostaining for γH2AX.

(B) U2OS cells transfected with either GFP-RNF8 or GFP-Empty for 2 h were then treated as in (A) and then subjected to western blotting for H2A, GFP and β-actin.

(C) Fibroblasts transfected with either GFP-RNF8 or GFP-Empty for 2 h were then treated as in (A), except that the immunostaining was for 53BP1.

(D) U2OS cells transfected with Flag-RNF8 or HA-RNF8 for 2 h were then treated as in (A) and immunostained for either Flag or HA and 53BP1. The white numbers indicate the percentage of 100 transfected i.e, green cells whose nuclei contained >10 IR-induced foci. Means ± SD are shown, n=3. * $p < 0.05$, as determined by a non-paired two-tailed Student's *t*-test..

Figure S5. Overexpression of 53BP1, MDC1 and RAD51 cannot restore repair foci and overexpression of RNF8 does not alter levels of repair factors in splicing-deficient cells

(A-C) U2OS cells were transfected with GFP-53BP1, GFP-MDC1 or GFP-RAD51 for 2 h, followed by addition of pladienolide B, isoginkgetin or DMSO, incubation for an additional 5 h, irradiation with 6 Gy, fixation 1 h later and immunostaining for (A) γH2AX or (B-C)

conjugated ubiquitin (FK2 antibody). The white numbers indicate the percentage of 100 transfected i.e, green cells whose nuclei contained >10 IR-induced foci. Means ± SD are shown, n=3. * $p < 0.05$, as determined by a non-paired two-tailed Student's *t*-test.

(D) U2OS cells were transfected with GFP-RNF168 for 2 h, either left untreated or irradiated with 6 Gy, fixated 1 h later and immunostained for γH2AX, FK2 or 53BP1.

(E) U2OS cells were transfected with GFP-RNF168 for 2 h, followed by addition of pladienolide B, isoginkgetin or DMSO, incubation for an additional 5 h, irradiation with 6 Gy where indicated, fixated 1 h later and immunostained for 53BP1.

(F) U2OS cells were transfected with either GFP-RNF8 or GFP-Empty for 2 h, followed by addition of DMSO, pladienolide B or isoginkgetin, incubation for an additional 5 h, irradiation with 6 Gy, harvested 24 h later, and subjected to western blotting for the indicated proteins.

Figure S6. Overexpression of RNF8 restores repair foci when these are impaired by depletion of splicing-related factors, but not when they are impaired by other means

(A) qPCR analysis of mRNA levels of the factors indicated in U2OS cells treated for 48 h with siRNAs targeting the factor itself. The change is relative to the two reference genes (18S rRNA and β-actin). Means ± SD are shown, n=3.

(B) qPCR analysis of RNF8 mRNA levels in U2OS cells treated with the siRNAs indicated for 48 h. The change is relative to the two reference genes (18S rRNA and β-actin). Means ± SD are shown, n=3.

(C) U2OS cells treated with the siRNAs indicated for 40 h, followed by transfection with GFP-RNF8 or GFP-Empty plasmids for 7 h, exposure to IR (6 Gy), fixation 1 h later and immunostaining for 53BP1.

(D) U2OS cells treated as in (C), except that the immunostaining was for FK2. The white numbers indicate the percentage of 100 transfected i.e, green cells whose nuclei contained >10 IR-induced foci. Means ± SD are shown, n=3. * $p < 0.05$, as determined by a non-paired two-tailed Student's *t*-test.

Supplementary Table S1. Primers used in this study

Primer name	Sequence 5'- 3'
MDC1 Forward (F)	5'-TGAACCTACCCAGGCCTTCATGTT-3'
MDC1 Reverse (R)	5'-ATTCAGGAGGCCTGTTGTCTGGAA-3'
WRAP53 F	5'-TGAAGACTTTGGAGACTCAACC-3'
WRAP53 R	5'-TATCAGCTCACCCACACCTC-3'
RNF8 F	5'-GATAGCCCAAGGAGAAAAGGA-3'
RNF8 R	5'-TTTGTTCTTGGCTTGAATGATT-3'
RNF168 F	5'-AGTGCAAGCTTAGAGCGTCTG-3'
RNF168 R	5'-TCTCTTCTCAGTTCCCCAGGT-3'
UBC F	5'-AGTAGTCCCTTCTCGGCGAT-3'
UBC R	5'-CACGAAGATCTGCATTGTCAAGT-3'
53BP1 F	5'-AGTGGTGAGAAACCAGTCAGTGCT-3'
53BP1 R	5'-TGACACGAGTGACAAGTGTGCGTA-3'
RAD51 F	5'-TTTGGAGAATTCCGAACTGG-3'
RAD 51 R	5'-CATCACTGCCAGAGAGACCA-3'
β-actin F	5'-AGGTCATCACCATTGCGAATGAG-3'
β-actin R	5'-CTTTGCGGATGTCCACGTCA-3'
18S rRNA F	5'-CGACGACCCATTCGAACGTCT-3'
18S rRNA R	5'-CTCTCCGGAATCGAACCCTGA-3'
H2AX F	5'-TACCTCACCGCTGAGATCCT-3'
H2AX R	5'-AGCTTGTTGAGCTCCTCGTC-3'
BRCA1 F	5'-TGTGCTTTTCAGCTTGACACAGG-3'
BRCA1 R	5'-CGTCTTTTGAGGTTGTATCCGCTG-3'
SF3B1 F	5'-CGCCAAGACTCACGAAGATATTG-3'
SF3B1 R	5'-GCCCACTCCTTGAGCTTCAT-3'

RBMX F	5’-CAGTTCGCAGTAGCAGTGGA-3’
RBMX R	5’-TCGAGGTGGACCTCCATAA-3’
PRPF8 F	5’-AACGCTCACCACCAAGGAAA-3’
PRPF8 R	5’-TGCACGTGACTATCCACCAC-3’
RNF8 exon1 F	5’-GGTGGCTGCTGCTGGAAGAT-3’
RNF8 exon2 R	5’-TGATACCAGTTGGTATGTGAC-3’
RNF8 intron1 R	5’-TGCCCTCCGCTCCACAGGA-3’
RNF8 exon7 F	5’-GCTCAGAAGTGAAAGAACGACG-3’
RNF8 intron7 F	5’-GAGGCTGGAAGCAGGGAGT-3’
RNF8 exon8 R	5’-AATGCCCTTAGAGCACGGTC-3’
RAD51 exon1 F	5’-TGCTGGAGAGAGGAGCGCT-3’
RAD51 intron1 R	5’-ACGTCCGGGTTTCACACTG-3’
RAD51 exon2 R	5’-GCCACACTGCTCTAACCGTG-3’
RAD51 exon9 F	5’-GGAAATATCATCGCCCATGCATC-3’
RAD51 intron9 F	5’-GCTCTGTTACAAAGTCAGGAACGG-3’
RAD51 exon10 R	5’-CAGGAAGACAGGGAGAGTCGTAG-3’
